# Reduced cell size, chromosomal aberration and altered proliferation rates are characteristics and confounding factors in the ST*Hdh* cell model of Huntington disease

**DOI:** 10.1038/s41598-017-17275-4

**Published:** 2017-12-04

**Authors:** Elisabeth Singer, Carolin Walter, Jonasz J. Weber, Ann-Christin Krahl, Ulrike A. Mau-Holzmann, Nadine Rischert, Olaf Riess, Laura E. Clemensson, Huu P. Nguyen

**Affiliations:** 1 Institute of Medical Genetics and Applied Genomics, University of Tuebingen, 72076 Tuebingen, Germany; 2 Centre for Rare Diseases, University of Tuebingen, 72076 Tuebingen, Germany; 3 Paediatric Haematology and Oncology, University Children’s Hospital Tuebingen, 72076 Tuebingen, Germany

## Abstract

Huntington disease is a fatal neurodegenerative disorder caused by a CAG repeat expansion in the gene encoding the huntingtin protein. Expression of the mutant protein disrupts various intracellular pathways and impairs overall cell function. In particular striatal neurons seem to be most vulnerable to mutant huntingtin-related changes. A well-known and commonly used model to study molecular aspects of Huntington disease are the striatum-derived ST*Hdh* cell lines generated from wild type and *huntingtin* knock-in mouse embryos. However, obvious morphological differences between wild type and mutant cell lines exist, which have rarely been described and might not have always been considered when designing experiments or interpreting results. Here, we demonstrate that ST*Hdh* cell lines display differences in cell size, proliferation rate and chromosomal content. While the chromosomal divergence is considered to be a result of the cells’ tumour characteristics, differences in size and proliferation, however, were confirmed in a second non-immortalized Huntington disease cell model. Importantly, our results further suggest that the reported phenotypes can confound other study outcomes and lead to false conclusions. Thus, careful experimental design and data analysis are advised when using these cell models.

## Introduction

Huntington disease (HD) is an inherited, fatal, neurodegenerative disorder. It results from a CAG repeat expansion in the gene *HTT*, coding for the huntingtin protein. The mutation is translated into an elongated polyglutamine repeat in huntingtin, which leads to the disruption of various cellular signalling pathways and results in impaired cell function and ultimately cell death, particularly of striatal neurons^1,2^. To study cellular and molecular mechanisms contributing to the HD pathogenesis, numerous cell and animal models have been generated. The ST*Hdh* cell lines were generated from an HD knock- in mouse model^3^, which carries the endogenous *Hdh* gene (mouse Huntington disease gene homolog) with a chimeric exon 1^4^ and is characterized by a mild behavioural phenotype and neuropathological features^5^. These cell lines derive from striatal primordia^3^ and express wild-type and mutant huntingtin at endogenous levels^6^. The precise genetic context and the striatal origin of the cells make the ST*Hdh* cell lines a widely used model in HD research. By comparing immortalized striatal precursor cells from wild type mice (ST*Hdh* ^Q7/Q7^ cells) to precursor cells derived from heterozygous and homozygous *Hdh* ^Q111^ knock-in mice (ST*Hdh* ^Q7/Q111^ and ST*Hdh* ^Q111/Q111^ cells), differences in a variety of HD-related cellular pathways have been discovered or confirmed, for instance an involvement of huntingtin in calcium handling deficits and mitochondrial dysfunction^7–11^ or effects on various signalling cascades^12–14^. Despite the to date unquestioned usefulness and importance of this model, obvious but rarely reported differences in size^11^, shape^15^ and proliferation rate might demand caution when using the ST*Hdh* cell lines. The origin of these differences, their importance for HD, as well as the consequences for the interpretation of study outcomes remains largely unaddressed.

In this study, we show that the ST*Hdh* cell lines exhibit divergent characteristics, which interfere with commonly used assays and hamper the direct comparison of both cell lines. We further show that these features are partially shared by mouse embryonic fibroblast (MEF*Hdh*) cell lines generated from the same animal model and their wild type littermates, which implies a common, HD-related mechanism beyond immortalization artefacts. Overall, these findings argue for a thorough characterization of every cell line used and the inclusion of such confounding factors in the experimental design.

## Results

### Reduced cell size is a characteristic of ST*Hdh*^Q111/Q111^ and MEF*Hdh*^Q111/Q111^ cells

**Figure 1:**
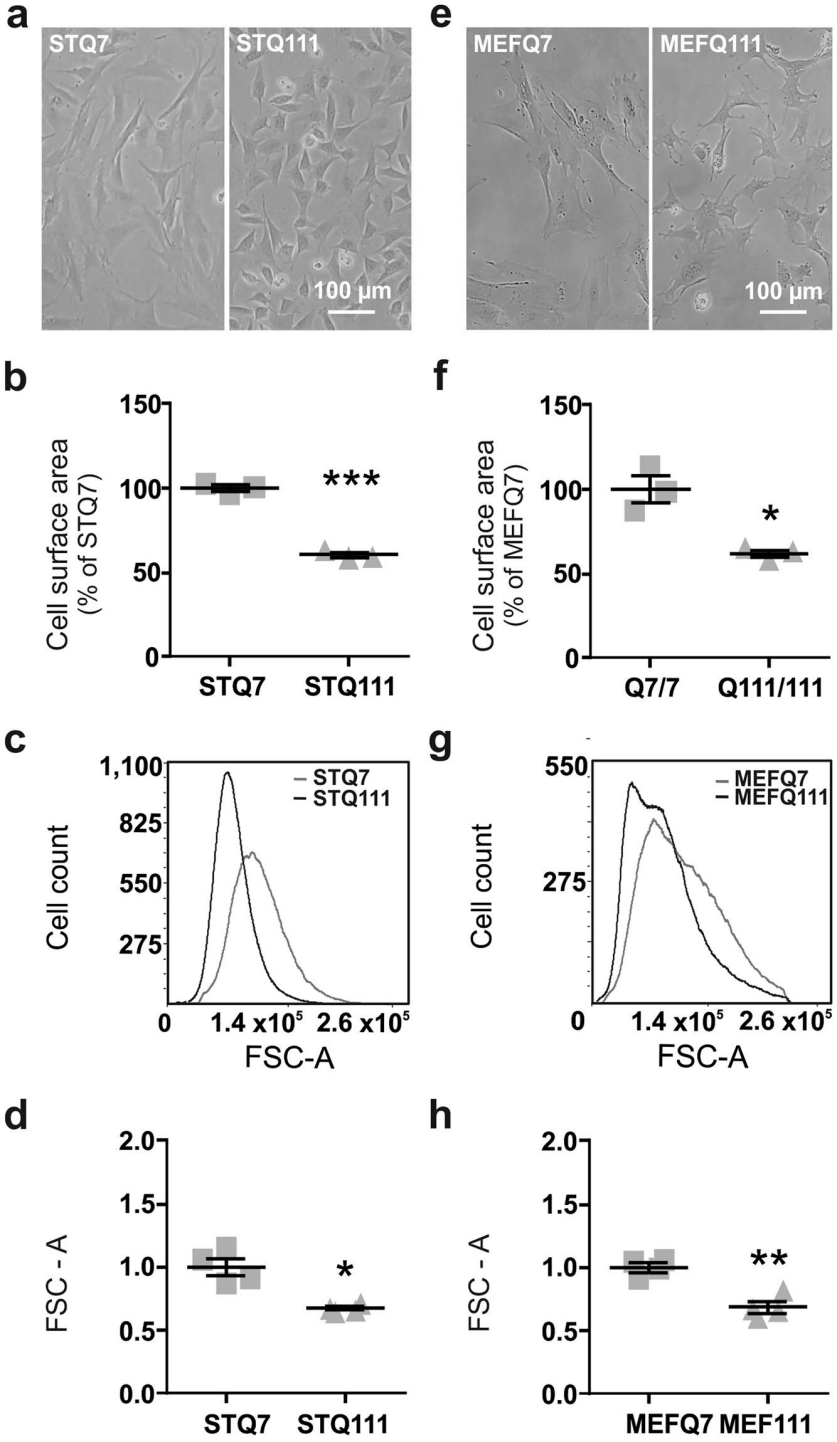
Cell size difference in Q111 knock-in cells. (**a**) Representative pictures of ST*Hdh* ^Q7/Q7^ (STQ7) and ST*Hdh* ^Q111/Q111^ (STQ111) cells, and (**b**) ImageJ-based surface area quantification of ST*Hdh* cells attached to the culture dish surface n = 3 experiments, unpaired *t*-tests; ***P < 0.001. (**c**) Representative histograms of ST*Hdh* cells and (**d**) quantification of the cell size of live cells in suspension, based on the relative mean forward scatter area (FSC-A); n = 4 experiments, unpaired *t*-tests; *P < 0.05. (**e–h**) Results of size determination for MEF*Hdh* ^Q7/Q7^ (MEFQ7) and MEF*Hdh* ^Q111/Q111^ (MEFQ111) cells, respectively; *P < 0.05, **P < 0.01.

We performed a morphometric analysis of homozygous ST*Hdh* ^Q111/Q111^ (STQ111) and wild type ST*Hdh* ^Q7/Q7^ (STQ7) cells by light microscopy and flow cytometry analysis. Measurement of the surface area of cells attached to the culture dish revealed a significantly smaller cell surface area in the mutant ST*Hdh* cells (Fig. 1a and b; *P* < 0.001). The smaller cell size of ST*Hdh* ^Q111/Q111^ was also found in detached cells, both when measuring the surface area from microscopic images (Supplementary Fig. S1) and on a larger scale by flow cytometry analysis (Fig. 1c and d). Here, the relative mean forward-scatter area (FSC-A), which is positively related to cell size, was 32% lower in ST*Hdh* ^Q111/Q111^ than in ST*Hdh* ^Q7/Q7^ cells (Fig. 1d; *P* = 0.013). Similar differences were also observed after differentiation into neuron-like cells (Supplementary Fig. S2).

To assess whether this cell size phenotype is cell line-specific or whether it might be considered a general feature of HD, we performed the same set of experiments in a fibroblast cell line established from the same mouse model (MEF*Hdh* cells). Like in the ST*Hdh* cells, the mutant MEF*Hdh* ^Q111/Q111^ (MEFQ111) cells had a smaller cell surface area compared to the wild type MEF*Hdh* ^Q7/Q7^ (MEFQ7) cells’, when the cells were attached to the culture dish (Fig. 1f; *P* = 0.03). Although the difference did not reach statistical significance when manually analysing cell surface area in detached cells (Supplementary Fig. S1; *P* = 0.13), it was detected again via flow cytometry analysis (Fig. 1g and h; *P* = 0.002). The relative mean FSC-A of MEF*Hdh* ^Q111/Q111^ cells was 31% lower compared to MEF*Hdh* ^Q7/Q7^ cells, comparable to the values retrieved for ST*Hdh* cells (Fig. 1h). Flow cytometry analysis further revealed a higher heterogeneity of the MEF*Hdh* cell population compared to ST*Hdh* cells, as represented by a broader distribution of cell sizes and two distinct peaks in the FSC-A plot (Fig. 1g), possibly due to the biological origin of these cell lines^16^.

### ST*Hdh* but not MEF*Hdh* cells show considerable chromosome abnormalities

As changes in DNA content can lead to alterations in cell size^17,18^ and are a common feature of cell line stabilization^19^ and cell passaging^20,21^, we performed a karyotype analysis to clarify whether the cell size differences observed in both cell lines are explained by changes in ploidy.

**Figure 2:**
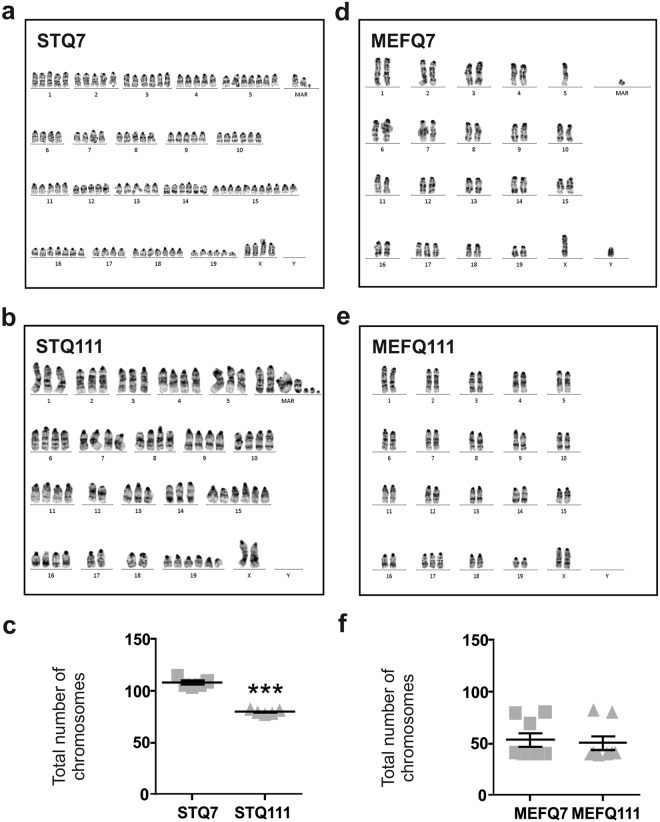
ST*Hdh* cells display marked and divergent chromosome abnormalities. (**a**) Representative karyograms from ST*Hdh* ^Q7/Q7^ (STQ7) and (**b**) ST*Hdh* ^Q111/Q111^ (STQ111) cells with **(c)** quantification of the chromosome numbers; n = 5 experiments, unpaired *t*-tests; ***P < 0.001. (**d–f**) Result of karyogram analysis for MEF*Hdh* ^Q7/Q7^ (MEFQ7) and MEF*Hdh* ^Q111/Q111^ (MEFQ111) cells, respectively; n = 8 experiments.

Karyotyping revealed a variety of chromosomal abnormalities in ST*Hdh* cells. Even more importantly, the chromosomal changes differed between ST*Hdh* ^Q111/Q111^ and ST*Hdh* ^Q7/Q7^ cells in qualitative and quantitative terms (Fig. 2a and b). In detail, ST*Hdh* ^Q7/Q7^ cells showed a hyperpentaploid, female, murine karyotype with chromosome numbers between 104 and 115. Different numerical anomalies as well as a variable number of additional, structurally abnormal chromosomes (three to eight marker chromosomes) were detected. About 40% of the cells showed at least one, but up to four additional copies of chromosome 3, 8, 9, 10, 14, 16 and 17. Interestingly, nearly 100% of the analysed cells showed two to six additional copies of chromosome 15, 18 and 19. Loss of at least one, but up to four copies was found for chromosome 4, 6, 7, 11, 12 and 13 in 40% of the cells. In contrast, ST*Hdh* ^Q111/Q111^ cells showed a hypo- to hypertetraploid, female, murine karyotype (77–82 chromosomes) with a high number (seven to nine) of marker chromosomes. Loss of one to four copies was found for chromosome 1, 4, 6, 7, 12, 14 and 18 - similar to ST*Hdh* ^Q7/Q7^ cells. Nearly all cells had one to three additional copies of chromosome 15 and 19. The total number of chromosomes was significantly lower in ST*Hdh* ^Q111/Q111^ cells compared to ST*Hdh* ^Q7/Q7^ cells (Fig. 2c; *P* < 0.001).

In contrast, MEF*Hdh* cells did not show marked chromosomal abnormalities (Fig. 2d and e). In detail, MEF*Hdh* ^Q7/Q7^ cells showed a mainly diploid, murine, male karyotype with only some tetraploid cells (Fig. 2d). Apart from a small number of single cell anomalies, no chromosomal losses were detected. A few cells showed additional copies of chromosome 16 and 17. MEF*Hdh* ^Q111/Q111^ cells showed a mainly diploid, female, murine karyotype and only a few tetraploid cells (Fig. 2e). Nearly all cells showed a numerically normal karyotype. About 50% of the cells were found to have an additional chromosome 17. The total number of chromosomes did not differ between MEF*Hdh* ^Q111/Q111^ and MEF*Hdh* ^Q7/Q7^ cells (Fig. 2f).

### ST*Hdh*^Q111/Q111^ and MEF*Hdh*^Q111/Q111^ cells show a higher proliferation rate

We further examined the proliferation rate of ST*Hdh* and MEF*Hdh* cells, as both mutant cell lines appeared to proliferate at different rates during regular passaging.

**Figure 3:**
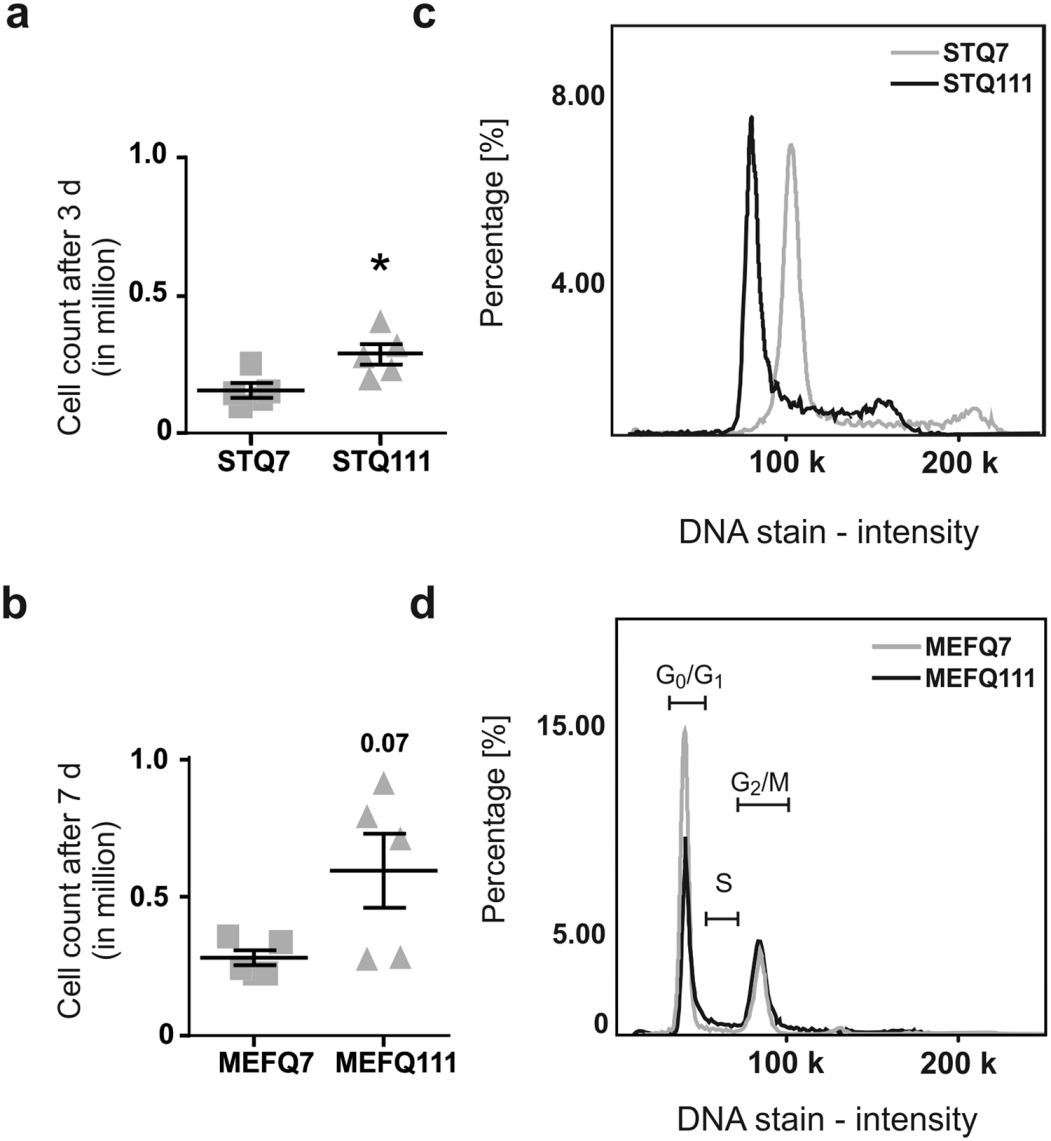
Both mutant cell lines exhibit increased proliferation rates. (**a**) Manually determined cell count of ST*Hdh* cells after 3 days; n = 5 experiments, unpaired *t*-tests; *P < 0.05 and (**b**) manually determined cell count of MEF*Hdh* cells after 7 days; n = 5 experiments; unpaired *t*-tests. (**c**) Representative overlays of signal intensity of ST*Hdh* ^Q7/Q7^ and ST*Hdh* ^Q111/Q111^ DAPI-stained cells and (**d**) representative overlay of signal intensity of MEF*Hdh* ^Q7/Q7^ and MEF*Hdh* ^Q111/Q111^ DAPI-stained cells with exemplary indication of cell cycle; n = 3.

Quantification of the increase in cell number after 3 days of cultivation revealed an elevated proliferation rate of ST*Hdh* ^Q111/Q111^ compared to ST*Hdh* ^Q7/Q7^ cells (Fig. 3a, *P* = 0.02). A trend towards increased proliferation rate was detected in MEF*Hdh* ^Q111/Q111^ compared to MEF*Hdh* ^Q7/Q7^ cells after 7 days of cultivation (Fig. 3b; *P* = 0.073), although both MEF*Hdh* cell lines did not proliferate as much as ST*Hdh* cells.

In order to clarify whether the enhanced proliferation had been the result of increased cell division or reduced cell death, we performed a cell cycle analysis and measured the amount of viable and apoptotic cells.

First, the proportion of cells in the different phases of the cell cycle was analysed by measuring the DNA content via DAPI staining intensity in detached, fixed cells. This assay confirmed the difference in ploidy between ST*Hdh* ^Q7/Q7^ and ST*Hdh* ^Q111/Q111^ cells, as there was a noticeable right shift in the curve obtained for ST*Hdh* ^Q7/Q7^ cells, corresponding to an overall increased DNA content (Fig. 3c). This shift, however, made the automated analysis by the analysis software unreliable, and was therefore not quantified. MEF*Hdh* cells, on the other hand and in line with their similar karyograms, exhibited similar distribution patterns of cell populations with different DNA content (Fig. 3d). In this case, the analysis showed a significant decrease in cells in the G_0_/G_1_ phase (MEF*Hdh* ^Q7/Q7^ 65.73 ± 2; MEF*Hdh* ^Q111/Q111^ 39.17 ± 1; *P* = 0.0003), alongside a tendency to an increase in cells in the S (MEF*Hdh* ^Q7/Q7^ 4.1 ± 2; MEF*Hdh* ^Q111/Q111^ 8.2 ± 0.2; *P* = 0.07) and G_2_/M phase (MEF*Hdh* ^Q7/Q7^ 22.9 ± 4; MEF*Hdh* ^Q111/Q111^ 38.1 ± 0.2; *P* = 0.03). The observed differences in cell cycle progression were in line with the observation that MEF*Hdh* cells containing the *huntingtin* knock-in mutation proliferate more than wild type cells.

**Figure 4:**
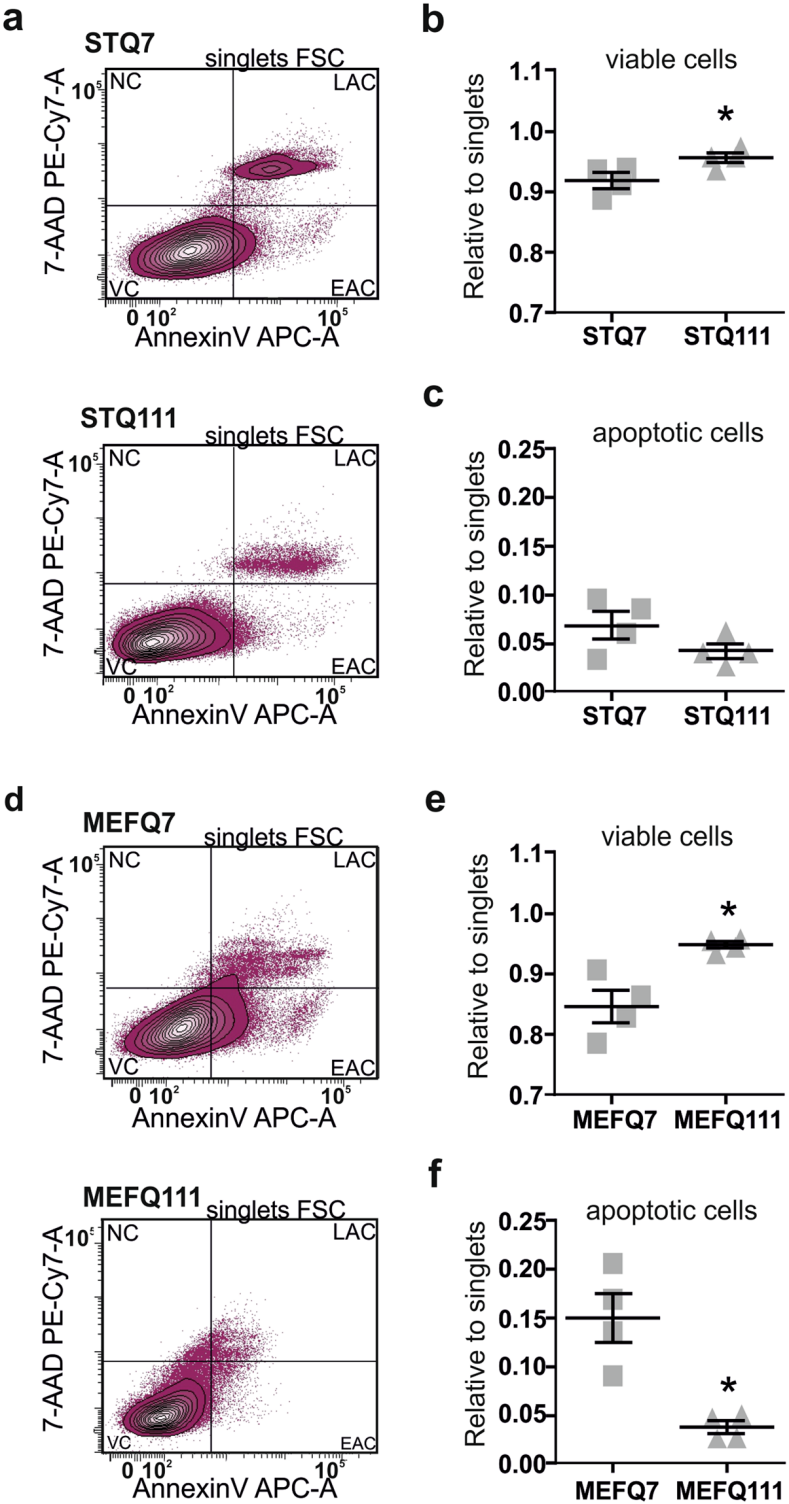
Cell viability is not reduced in ST*Hdh* and MEF*Hdh* mutant cell lines. Results from cell size- and cell number-independent flow cytometry analysis: (**a**) Representative scatterplots of flow cytometry analysis of ST*Hdh* cells and (**b** and **c**) quantification from flow cytometry analysis of Annexin V/7′AAD staining; n = 4 experiments. VC: viable cells, EAC: early apoptotic cells, LAC: late apoptotic cells, NC: necrotic cells. Quantification of apoptotic cells combines results for EAC and LAC; unpaired *t*-tests; *P < 0.05. (**d–f**) Results of MEF*Hdh* cells, respectively; unpaired *t*-tests; *P < 0.05.

Second, we analysed the amount of viable and apoptotic cells by flow cytometry analysis (Fig. 4). We found ST*Hdh* ^Q111/Q111^ cells to have a higher proportion of viable cells (Fig. 4b, *P* = 0.047), and in turn a lower proportion of apoptotic cells compared to ST*Hdh* ^Q7/Q7^ cells, although the latter did not reach statistical significance. Similar results were obtained for MEF*Hdh* cells, showing a significantly higher proportion of viable cells (Fig. 4e; *P* = 0.026) and, in this case, a significantly lower number of apoptotic cells (Fig. 4f; *P* = 0.017) in MEF*Hdh* ^Q111/Q111^ cells compared to their wild type control.

### The cell size and proliferation phenotypes in ST*Hdh*^Q111/Q111^ cells might impede the interpretation of standard cell viability assays

**Figure 5:**
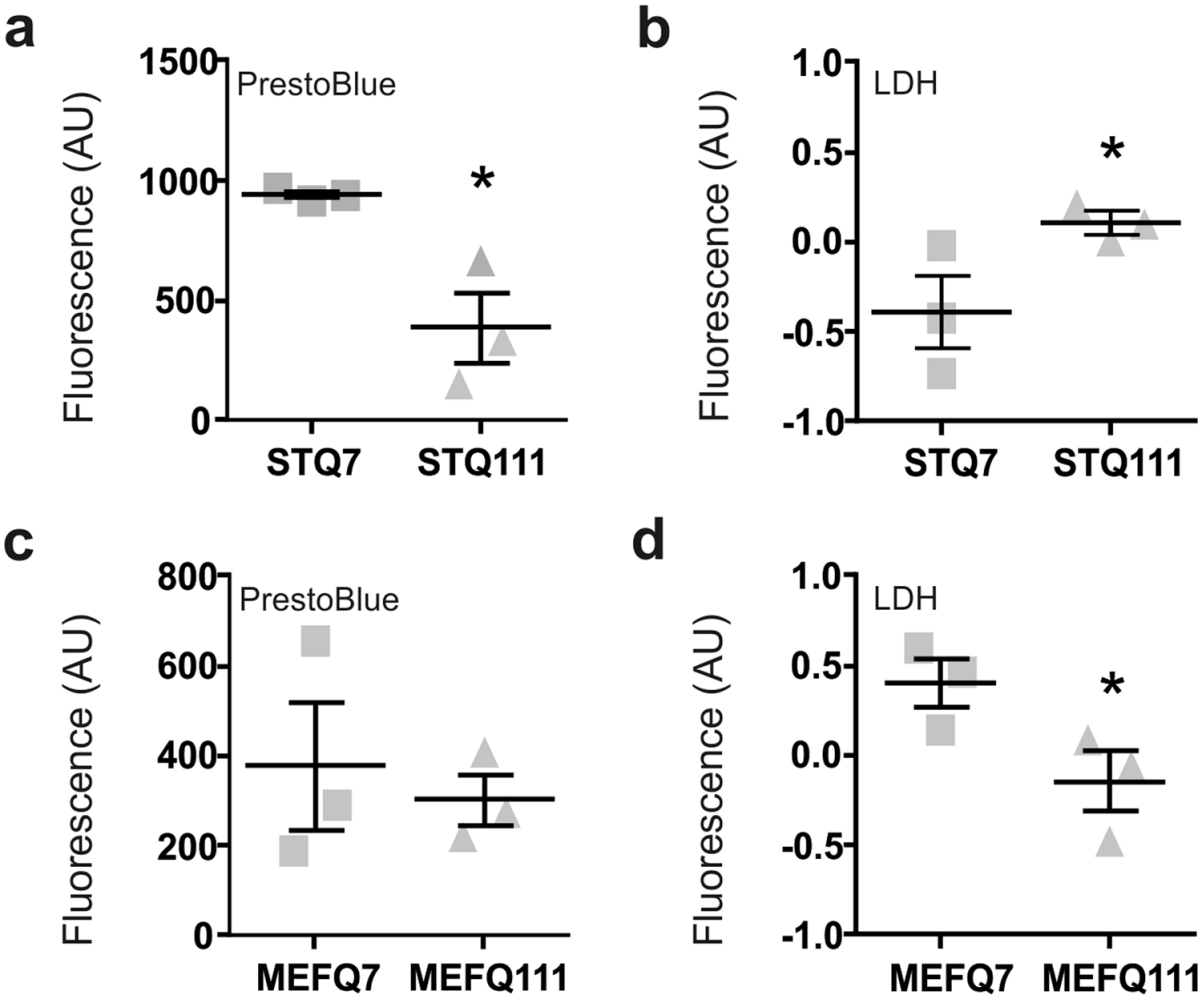
The cell size and proliferation phenotypes in ST*Hdh* ^Q111/Q111^ cells impede the interpretation of standard cell viability assays. Results from the cell size- and cell number-dependent tests for ST*Hdh* cells: (**a**) PrestoBlue^®^, n = 3 experiments and (**b**) LDH assay, n = 3 experiments. Unpaired *t*-tests; *P < 0.05. (**c** and **d**) Results from size- and cell number-dependent tests for MEF*Hdh* cells, respectively; unpaired *t*-tests; *P < 0.05.

When investigating cell viability in our study, we used flow cytometry, a method that should theoretically be independent of cell size and cell proliferation. However, common cell viability tests depend considerably on these parameters. Thus, we reassessed cell viability and cell death using the standard cell viability assays, PrestoBlue^®^ and LDH assay, respectively (Fig. 5).

Analysis of the data revealed contradicting results when compared to the outcomes from flow cytometry. The PrestoBlue^®^ assay consistently showed lower signals in ST*Hdh* ^Q111/Q111^ cells (Fig. 5a; *P* = 0.031) and the LDH assay revealed increased LDH release in ST*Hdh* ^Q111/Q111^ compared to ST*Hdh* ^Q7/Q7^ cells (Fig. 5b; *P* = 0.022), suggesting that mutant cells are characterized by reduced viability and increased cell death, in contrast to the first findings. Differentiation of ST*Hdh* cells led to a similar readout as flow cytometry (Supplementary Fig. S3).

The results obtained for MEF*Hdh* cells differed from the results obtained for ST*Hdh* cells. MEF*Hdh* ^Q111/Q111^ cells had similar signals as MEF*Hdh* ^Q7/Q7^ cells in the PrestoBlue^®^ assay (Fig. 5c; *P* = 0.656), but showed reduced LDH release (Fig. 5d; *P* = 0.034). These findings were comparable to the results obtained by flow cytometry.

### Chromosomal abnormalities in ST*Hdh*^Q111/Q111^ cells might impede the interpretation of western blot analyses

Since we observed that ST*Hdh* ^Q111/Q111^ cells differ markedly from the control ST*Hdh* ^Q7/Q7^ cell line in terms of chromosomal constitution, we investigated possible consequences of these alterations on the protein levels of commonly used loading controls for western blot analysis. The four proteins, β-actin (*Actb*, chromosome 5), GAPDH (*Gapdh*, chromosome 6), α-tubulin (*Tuba1a*, chromosome 15) and vinculin (*Vcl*, chromosome 14) are located on different chromosomes.

**Figure 6:**
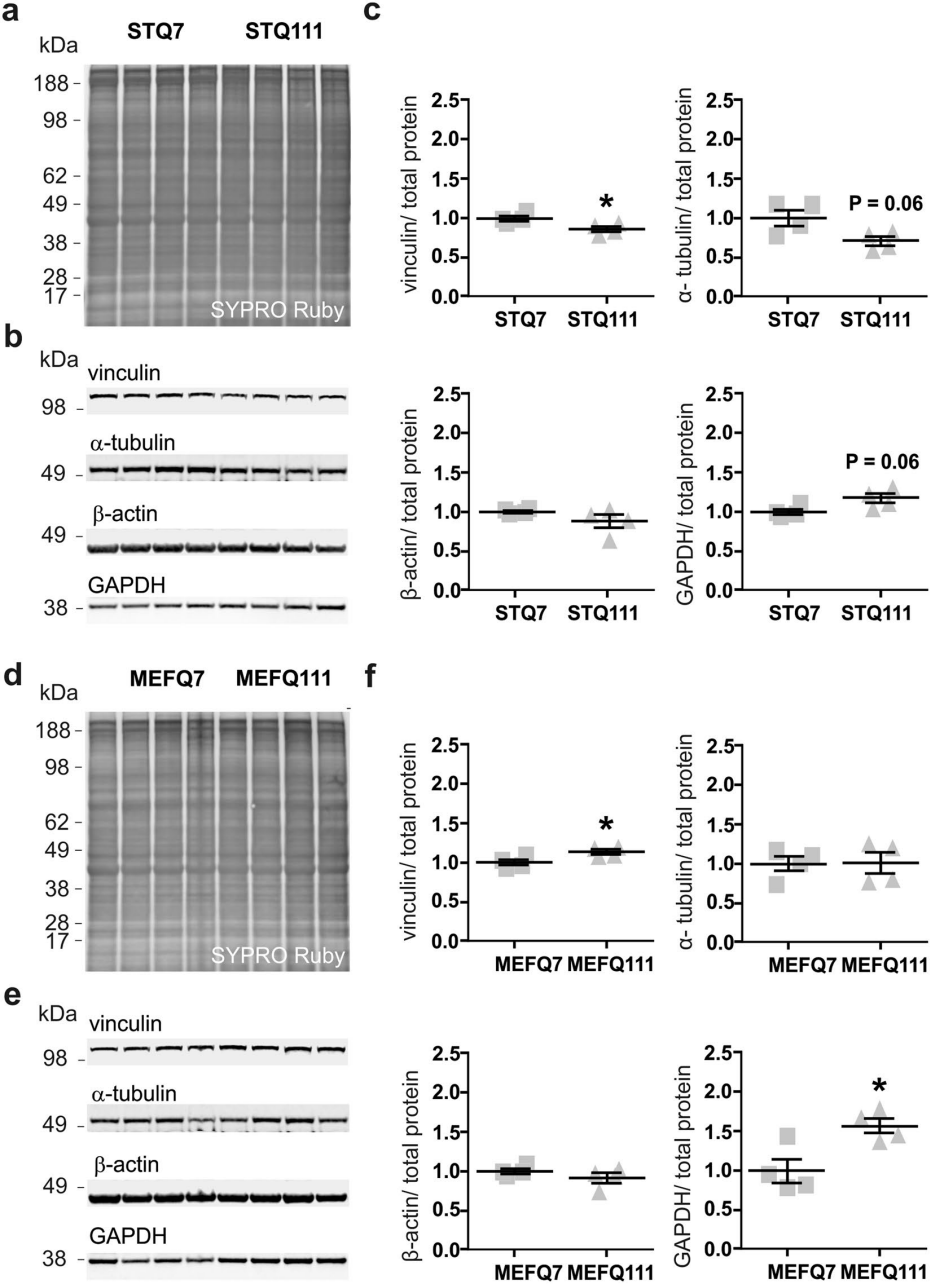
Chromosomal abnormalities impede the interpretation of western blot results in ST*Hdh* cells. (**a**) SYPRO Ruby staining, (**b**) western blots and (**c**) corresponding quantification of marker proteins in ST*Hdh* cells. Unpaired *t*-tests; *P < 0.05. (**d**) SYPRO Ruby staining, (**e**) western blots and (**f**) corresponding quantification of marker proteins in MEF*Hdh* cells. Unpaired *t*-tests; *P < 0.05.

Western blot analysis of RIPA cell lysates revealed strong trends toward decreased levels of α-tubulin and vinculin in ST*Hdh* ^Q111/Q111^ cells compared to ST*Hdh* ^Q7/Q7^ cells (Fig. 6b and c; *P* = 0.06, *P* = 0.03), in accordance with the reduced number of chromosomes 15 and 14 in ST*Hdh* ^Q111/Q111^ cells. In contrast, these differences were not found in MEF*Hdh* cells (Fig. 6e and f), where no differences in the number of chromosomes 15 and 14 were detected. Furthermore, the levels of β-actin were comparable in ST*Hdh* ^Q111/Q111^ and ST*Hdh* ^Q7/Q7^ cells (Fig. 6b and c) as well as in MEF*Hdh* ^Q111/Q111^ and MEF*Hdh* ^Q7/Q7^ cells (Fig. 6e and f), in accordance with the similar numbers of chromosome 5 in mutant and control cell lines. Interestingly, despite equal numbers of chromosome 6, levels of GAPDH were elevated in ST*Hdh* ^Q111/Q111^ and tendentially in MEF*Hdh* ^Q111/Q111^ cell lines, compared to their wild type counterparts (Fig. 6b,c; *P* = 0.06, e and f; *P* = 0.02).

## Discussion

ST*Hdh* cells represent a widely used cell culture model for studying cellular and molecular aspects of HD. Differences in cell morphology, growth and differentiation have previously been mentioned by other groups^15,22^, but to date, these differences have not been assessed quantitatively. Our study demonstrates clear differences in cell size, proliferation and ploidy between mutant and wild type ST*Hdh* cells, and suggests a strong influence of these phenotypes on other readouts.

In the first description of the ST*Hdh* cell lines, it was stated that ST*Hdh* ^Q111/Q111^ cells are of similar size as ST*Hdh* ^Q7/Q7^ cells, while cell proliferation was even decreased in the mutant cells and accompanied by an increase in DNA content^3^. Later studies, however, either do mention a reduced cell size of ST*Hdh* ^Q111/Q111^ cells^11,15^, or the results are at least suggestive of such a phenotype (although not specifically discussed in these papers)^23–25^. This might indicate that the phenotypes observed in our study had developed over time, possibly due to the tumour character of the cell lines. On the other hand, a reduced cell size was also found in our MEF*Hdh* ^Q111/Q111^ cell line compared to the respective control, despite the absence of large scale chromosomal changes. In addition, cell size differences in striatal neurons have been reported for the R6/2 model^26,27^ and the YAC128 model^28^, two transgenic mouse models of HD, and it has been suspected for HD patients^29^. It remains uncertain, if the reduced cell size should be considered an artefact or could be an HD-related feature, although it might be concluded that huntingtin is at least somehow involved in cell size regulation, as it is, as well, known to interact with cytoskeletal proteins^30^.

The multiple numerical anomalies and structurally abnormal chromosomes we found in both ST*Hdh* cell lines are typical for stable cell lines and long-term passaging^19–21^. Importantly, these abnormalities were found in cell populations that had been passaged for a maximum of six times between their purchase and the respective karyogram analysis. As this is a normal amount of passages required to carry out experiments, the abnormalities are likely to appear in other laboratories in a similar magnitude. Thus, users should be aware that the cell lines might not show the characteristics according to the original publication.

We further found the ST*Hdh* ^Q111/Q111^ as well as MEF*Hdh* ^Q111/Q111^ cells to have an increased proliferation rate. It had been reported earlier that mutant huntingtin is involved in cell division in cell models and *Drosophila* ^31^, as well as *Hdh* ^Q111/111^ knock-in mice, ST*Hdh* ^Q111/Q111^ cells and MEF*Hdh* ^Q111/Q111^ cells, as it alters the orientation of the mitotic spindle^32^. Although cell proliferation had not been measured in that study, the authors demonstrate that this leads to changes in neurogenesis in the developing cortex, highlighting the importance of this phenotype.

It is perceivable that differences in cell size, proliferation rate and chromosomal content might constitute confounding factors, and might complicate the interpretation of study outcomes due to adding several variables which cannot properly be controlled for. We demonstrated that assays based on cell size and number, such as the PrestoBlue^®^ and LDH assay, revealed lower basal cell viability and increased cell mortality in ST*Hdh* ^Q111/Q111^ cells. Similar results have previously been published by others using the same assays^33–35^ or comparable methods^7^. However, the results could not be recreated in an assay that was likely to not depend on cell size or cell number. Thus, the earlier reported baseline difference in cell viability between ST*Hdh* ^Q111/Q111^ and ST*Hdh* ^Q7/Q7^ cells is questionable. Interestingly, our results were even indicative of increased cell viability in both, ST*Hdh* ^Q111/Q111^ and MEF*Hdh* ^Q111/Q111^. Effects on pro-survival functions in ST*Hdh* ^Q111/Q111^ cells would need to be further investigated, as they have been reported to be reduced for other cell models of HD^36,37^, whereas Akt signalling, implicated in neuronal survival^38^, has been shown to be increased in mutant ST*Hdh* cells^14^. Clearly, ST*Hdh* cells do not represent the hallmarks of the advanced disease. Intranuclear inclusions, amongst others, found in *in vitro* and *in vivo* models, are not found in ST*Hdh* cells^3^. Therefore, the disease stage they model might not necessarily be characterized by a reduction in cell viability under normal conditions.

Confounding effects of the chromosomal abnormalities found in the ST*Hdh* cell lines were further expected for western blot analyses. Our investigations revealed important aspects to be considered when choosing a loading control for western blot analysis in ST*Hdh* cells. The protein levels of α-tubulin and vinculin were lower in ST*Hdh* ^Q111/Q111^, but not in MEF*Hdh* ^Q111/Q111^, when compared to their respective controls and can be interpreted as a direct effect of the lower copy number of the chromosomes 14 and 15 in the ST*Hdh* ^Q111/Q111^ cells. These observations correspond to previous studies, which reported on analogous proteomic changes resulting from variations of the gene copy number in cancer cells or aneuploid cell lines^39,40^. On the other hand, our observation of an elevated GAPDH expression in both ST*Hdh* ^Q111/Q111^ and MEF*Hdh* ^Q111/Q111^ cells has already been shown in other HD models. As GAPDH is a well-known interaction partner of huntingtin, these results further render GAPDH as an inadequate loading control in HD research^41–43^.

Although our study is important, as it demonstrates features of the extensively used ST*Hdh* model that need to be considered when working with this cell model, and as it highlights the MEF*Hdh* cells as useful controls in *in vitro* studies, there are some limitations that we would like to point out. First, our MEF*Hdh* cells were generated from embryos of different sex. The MEF*Hdh* cells were generated 12 days after a 48-hour breeding period, and sex differentiation in the mouse embryo begins as early as E10^44^. Therefore, although we consider the influence of sex determination on cell size, proliferation rate and chromosomal content at that point negligible, we cannot rule it out. Thus, we highly recommend the generation of sex-matched MEF*Hdh* cell lines for further studies. Second, the two cell lines characterized here originate from the same HD animal model. As such, they share several drawbacks that need to be considered. *Hdh* ^Q111^ knock-in mice, like most other animal models of HD, are designed to express mutant huntingtin with high numbers of polyglutamine repeats to provoke possibly early and strong phenotypes (reviewed by Ferrante *et al*.^45^), even though such high repeat numbers are only found in patients with the rare juvenile form of HD. In this regard, it should be noted that cell models^46^ and animal models^47^ with lower CAG-repeats have been generated to recapitulate the commonly found mutation lengths. Furthermore, both ST*Hdh* and MEF*Hdh* cell lines are not isogenic. Q7 alleles represent the wild type mouse alleles, while Q111 alleles are human mouse chimera of exon1. For this reason, there are additional differences in the gene sequence between Q7 and Q111 alleles than the CAG repeat expansion. On the other hand, ST*Hdh* and MEF*Hdh* cells differ fundamentally regarding immortalization and biological origin. ST*Hdh* cells are comparable to other immortalized cell lines with regard to immortalization artefacts^19–21,48^, as shown here by the altered chromosome numbers. This is a drawback, as the supposedly complementary Q7 and Q111 cell lines have apparently acquired divergent features over time. Moreover, it needs to be considered that p53, a tumour suppressor protein affected in immortalized cell lines^49,50^, is a transcriptional regulator of *huntingtin* ^51^ and implicated in the pathogenesis of HD^52^. In this regard, the MEF*Hdh* cells used here represent a better cell model, as these were not immortalized and therefore the genetic integrity was less corrupted. However, the MEF*Hdh* cells presented milder phenotypes regarding cell size and proliferation, which is likely to be due to their heterogeneous cell composition^16^. In this regard, the clonal and neuronal character of ST*Hdh* cells might lead to stronger and more robust phenotypes than embryonic fibroblasts. The clonal character, however, once more underscores the importance of an additional model, to exclude artefacts. Finally, it would always be advantageous to confirm phenotypes in cell and animal models of HD that are based on a different genetic background.

In summary, ST*Hdh* cell lines are a generally useful model to study mechanisms behind the molecular pathogenesis of HD, because they provide the proper cellular as well as genetic context of HD due to their striatal origin and the knock-in model they derive from. However, the possible bias due to differences in cell size, proliferation and chromosomal content need to be considered when planning and interpreting results. In this regard, assays in which cell size and cell number play an important role for the outcome, and cannot be controlled for, should be avoided. Differentiation of the ST*Hdh* cells into neuron-like cells might at least overcome the problem regarding cell proliferation. Nevertheless, for time-course experiments the increased proliferation rate, as it was, as well, observed in MEF*Hdh* cells needs to be considered. A simple solution for treatment studies would be to not directly compare results from ST*Hdh* ^Q7/Q7^ to ST*Hdh* ^Q111/Q111^, but to rather compare treatment effects in the two cell lines independently. Finally, using a second *in vitro* or an *in vivo* model to confirm results is beneficial to determine the HD-dependency of the phenotype investigated. Our study emphasizes that it is of importance to regularly check the basic characteristics of an employed cell model and to consider putative alterations for experimental design and analysis.

## Methods

### Ethics Statement

Experiments for the generation of MEF*Hdh* cells were performed at the University of Tuebingen. The protocol was approved by the local ethics committee at Regierungspraesidium Tuebingen and carried out in accordance with the German Animal Welfare Act and the guidelines of the Federation of European Laboratory Animal Science Associations, based on European Union legislation (Directive 2010/63/EU).

### ST*Hdh* cells

ST*Hdh* cell lines, originally generated at the laboratory of Dr. Marcy MacDonald (Harvard Medical School, Boston)^3^, were purchased from Coriell Cell Repositories (Coriell Institute for Medical Research). Cell passages 4–12 were used for the experiments.

### MEF*Hdh* cells

A heterozygous breeding of *Hdh* ^Q111^ knock-in mice was set up and maintained for 48 hours. After 12 days, the pregnant female was sacrificed by inhalation of CO_2_. The embryos were extracted by caesarean sectioning, decapitated immediately and placed individually in sterile, ice-cold, Dulbecco’s phosphate-buffered saline (DPBS) (Invitrogen). Limbs, brain and visceral organs were removed. The remaining tissue was transferred into a sterile well of a 6-well plate with fresh DPBS, which was then replaced by 2 ml of culture media (Dulbecco’s Modified Eagle Medium (DMEM) with 1% penicillin/streptomycin and 10% fetal calf serum (FCS), Gibco^®^, Thermo Fisher Scientific). The tissue was incubated for 1 h at 37 °C and 5% CO_2_. After this, the tissue was transferred into a 100 mm dish with 10 ml culture media (pre-warmed to 37 °C), and minced with a scalpel. Pieces were transferred to a T75 cell culture flask with 10 ml of fresh media and incubated at 37 °C and 5% CO_2_ for 3 days. Afterwards, media was changed and the cells were incubated until they reached 90% confluency. Cells were then trypsinized (1 ml 0.25% trypsin-EDTA (Invitrogen) for 5 min at 37 °C and 5% CO_2_) and gently resuspended using a 1 ml pipette for subcultivation. For the experiments, a wild type and a homozygous culture were picked.

### Cell handling and treatment

ST*Hdh* and MEF*Hdh* cells were maintained in DMEM supplemented with 10% FCS (Gibco^TM^) and 1% penicillin/streptomycin (Gibco^TM^) at 37 °C in 5% CO_2_. ST*Hdh* media was additionally complemented by adding 1% geneticin (A2912, Biochrome). Both, ST*Hdh* and MEF*Hdh* cells were routinely tested negative for contamination by mycoplasma using the Venor®*GeM* Mycoplasma detection kit (Merck). Unless specifically stated differently, ST*Hdh* cells were undifferentiated. For differentiation into neuron-like cells a previously described differentiation protocol^3^ was used. For this, cells were incubated in differentiation cocktail for 24–48 h.

### Flow cytometry

Undifferentiated ST*Hdh* and MEF*Hdh* cells were recorded using a flow cytometry LSR II cytofluorometer (BD Bioscience). A total of 200,000 ungated events were analysed with the flow cytometry-DIVA software version 6.1.3 (BD Bioscience) and overlays were processed with FCS Express software version 4.0.230 (De Novo Software). Differentiated ST*Hdh* cells were recorded with a CyAn™ ADP flow cytometer (Beckman Coulter). A total of 20,000 ungated events were analysed with Summit V4.3.01 software (Dako Colorado, Inc.).

### Cell size determination

Cells were seeded in 6-well plates and grown to 60–70% confluency. Cell size was measured for cells attached to the surface of the culture dish as well as for detached cells after trypsinization each with 3 replicates per cell line. A total of 270 cells per genotype were analysed in 3 independent experiments (30 cells/well; 3 wells/experiment). Pictures of the cells were taken using an Eclipse TS100 Inverted Routine microscope (Nikon) with a digital camera at 20x magnification and analysed with ImageJ v1.47^53^. For attached cells, the area of the cells was approximated by measuring the area of a polygon that was assigned to each cell. For detached cells, the area of a round shape was measured that was applied to each cell individually. The scale was determined by the length of the counting chamber grid.

### Chromosome analysis

Chromosome preparations from cultured cells and GTG-banding were performed using standard techniques. For each cell line, 17 mitoses were numerically analysed and 5–8 mitoses were structurally analysed. For cytogenetic analyses, for all cell lines, cells from early passages (P4-P6) were harvested using a standard protocol and was followed by G-banding. Images of well spread metaphase chromosomes were captured using a CCD camera. Karyotyping was performed using the IKAROS software (MetaSystems, Altlussheim, Germany). Chromosome classification followed the guidelines of the International Committee on Standardized Genetics nomenclature for mice^54^.

### Determination of proliferation rate

Three replicates of ST*Hdh* (40,000 cells per well) and MEF*Hdh* cells (100,000 cells per well) were seeded in 6-well plates. After 3 days (ST*Hdh*) or 7 days (MEF*Hdh*), cells were harvested by trypsinization (250 µl 0.25% trypsin-EDTA (Gibco^TM^) for 5 min at 37 °C and 5% CO_2_), washed and counted again. At least three independent experiments were performed.

### Determination of DNA content

DNA content was measured using the NucleoView NC-3000 (ChemoMetec). Reagents were provided by the manufacturer and cells were treated according to the manufacturer’s instructions. In brief, cells were detached from the culture flask, washed with DPBS (Gibco^TM^) and lysed. The cells were stained with DAPI, at a saturating concentration (10 µg/ml), stabilized and immediately analysed with the device. Data was analysed with the NucleoView NC-3000 software, Version 2.1.25.12 (ChemoMetec).

### Viability assays

Cell viability and cell death were determined using commercially available kits (PrestoBlue^®^ cell viability reagent, Invitrogen^TM^; Cytotoxicity Detection Kit (LDH), Roche), following the provider’s instructions. Briefly, 10,000 cells were seeded in a 96-well plate and incubated overnight. Culture media was transferred into a new 96 well plate for the LDH assay. Cells left in the original plate received fresh media containing PrestoBlue^®^. The fluorescence intensity (PrestoBlue^®^ assay) was measured after 1 h; the absorption (LDH assay) was measured according to manufacturer’s instructions, using the plate reader MWGt Synergy HT (BioTek Instruments) and the software Gen5 2.01 (BioTek).

In addition, cell viability and cell death were measured using flow cytometry. For this, cells were grown in 75 ml culture flasks and harvested by gentle trypsinization (0.25% Trypsin-EDTA; Gibco^®^, Thermo Fisher Scientific). Cells were centrifuged at 400 × *g* for 5 min and washed twice with 1× Annexin V Binding Buffer (eBioscience). Cells were labelled with Alexa Fluor® 647 Annexin V (Biolegend) and 7-Amino-Actinomycin (7′AAD) (BD Pharmingen). Data was recorded by flow cytometry to determine the number of Annexin V/7′AAD-positive cells.

### Cell lysate preparation

For preparation of lysates, ST*Hdh* and MEF*Hdh* cells were trypsinized and collected by centrifugation at 350 × *g* for 5 min. The pellet was washed once with cold DPBS (Gibco^®^, Thermo Fisher Scientific), centrifuged again and lysed in RIPA buffer (50 mM Tris pH 7.5, 150 mM NaCl, 0.1% SDS, 0.5% sodium deoxycholate and 1% Triton X-100, containing protease inhibitors) for 25 min on ice, while vortexing briefly every 5 min. Afterwards, samples were centrifuged at 13,200 × *g* for 15 min at 4 °C. Supernatant was pipetted into a pre-cooled reaction tube, adding glycerol to final concentration of 10%, and stored at −80 °C until further analysis.

### Western blotting, SYPRO Ruby staining and immunodetection

Protein concentrations of RIPA lysates were determined spectrophotometrically using Bradford reagent (Bio-Rad Laboratories). Western blot analysis was performed following standard procedures. Briefly, 30 µg of protein were separated electrophoretically using 10% Bolt® Bis-Tris Plus Gels (Thermo Fisher Scientific). Proteins were transferred on Amersham™ Protran™ Premium 0.2 µm nitrocellulose membranes (GE Healthcare) using a TE22 Transfer Tank (Hoefer).

After transfer, total protein was stained with SYPRO Ruby Protein Blot Stain (Thermo Fisher Scientific) according to manufacturer’s instructions and detected at 600 nm using the LI-COR ODYSSEY^®^ FC imaging system (LI-COR Biosciences).

After SYPRO Ruby staining, membranes were blocked with 5% Slimfast in TBS at room temperature for 1 h and probed overnight at 4 °C with the following primary antibodies: mouse anti-β-actin (1:10.000; clone AC-15, A5441, Sigma Aldrich), mouse anti-GAPDH (1:1000; clone GA1R, ab125247, Abcam), mouse anti-α-tubulin (1:5000; clone DM1A, CP06, EMD Millipore) and rabbit anti-vinculin (1:1000; clone E1E9V, #13901, Cell signalling). Afterwards, membranes were incubated at room temperature for 1 h with the respective secondary IRDye antibodies goat anti-mouse 680LT and goat anti-rabbit 800CW (all 1:10,000; LI-COR Biosciences). Fluorescence signals were detected with the LI-COR ODYSSEY^®^ FC and quantified with ODYSSEY^®^ Server software version 4.1 (LI-COR Biosciences). Quantified signals were normalized to total protein as detected before using SYPRO Ruby Protein Stain.

### Statistical Analysis and Data availability

All data are presented as individual measurements (grey shapes) with mean and standard error of the mean (SEM). Statistical analysis was performed with GraphPad Prism 6.00 for Windows (GraphPad Software, Inc). Statistical significance of two group data sets was determined using two- tailed, unpaired Student’s *t*-test, with Welch’s correction. The significance threshold was set to *P* < 0.05. The datasets generated during and/or analysed during the current study are available from the corresponding author on reasonable request.

## Electronic supplementary material

Supplementary Information
