# Supplementary material for: Reduced cell size, chromosomal aberration and altered proliferation rates are characteristics and confounding factors in the STHdh cell model of Huntington disease
Source: Sci Rep. 2017 Dec 4;7:16880. doi: 10.1038/s41598-017-17275-4 (PMC5715050; doi:10.1038/s41598-017-17275-4)
Supplement: Supplementary file 1 — Supplementary Information [file 41598_2017_17275_MOESM1_ESM.pdf]

Supplementary Figures

Reduced cell size, chromosomal aberration and altered proliferation rates are characteristics and confounding factors in the STHdh cell model of Huntington disease

Elisabeth Singer[1,2,#], Carolin Walter[1,2,#], Jonasz J. Weber[1,2,#], Ann-Christin Krahl[3], Ulrike A. Mau-Holzmann[1], Nadine Rischert[1,2], Olaf Riess[1,2], Laura E. Clemensson[1,2,#] and Huu P. Nguyen[1,2,#,*]

1 Institute of Medical Genetics and Applied Genomics, University of Tuebingen, 72076 Tuebingen, Germany

2 Centre for Rare Diseases, University of Tuebingen, 72076 Tuebingen, Germany

3 Paediatric Hematology and Oncology, University Children's Hospital Tuebingen, 72076 Tuebingen, Germany.

# These authors contributed equally

* Corresponding author: Dr. Huu Phuc Nguyen, Institute of Medical Genetics and Applied Genomics, Centre for Rare Diseases, University of Tuebingen, Calwerstrasse 7, Tuebingen, Germany, Phone: +49 7071 29 83210, Fax: +49 7071 29 5228, Email: hoa.nguyen@med.uni-tuebingen.de

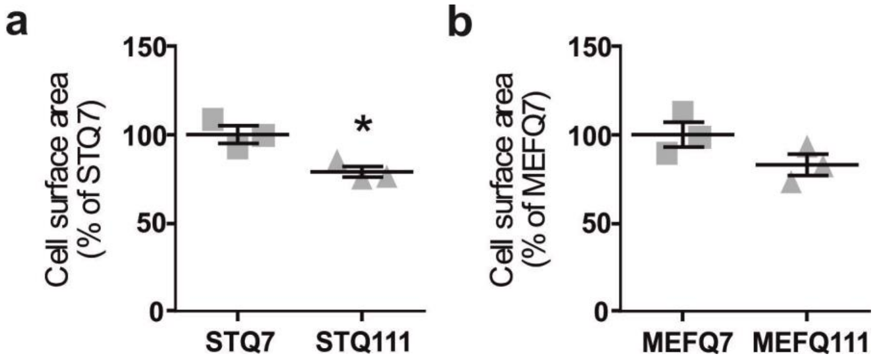

Supplementary Figure S1: Cell size differences of detached cells can be manually determined in ST*Hdh* cells

(a) ImageJ-based cell surface area quantification of ST*Hdh*$^{Q7/Q7}$ (STQ7) and ST*Hdh*$^{Q111/Q111}$ (STQ111) cells, detached from the culture dish by gentle trypzination, taken from cell suspension loaded to a Neubauer chamber; n = 3 experiments, each with 3 replicates per cell line, unpaired *t*-tests; * $P < 0.05$ **(b)** ImageJ-based cell surface area quantification of detached MEF*Hdh*$^{Q7/Q7}$ (MEFQ7) and MEF*Hdh*$^{Q111/Q111}$ (MEFQ111) cells; n = 3 experiments, unpaired *t*-test.

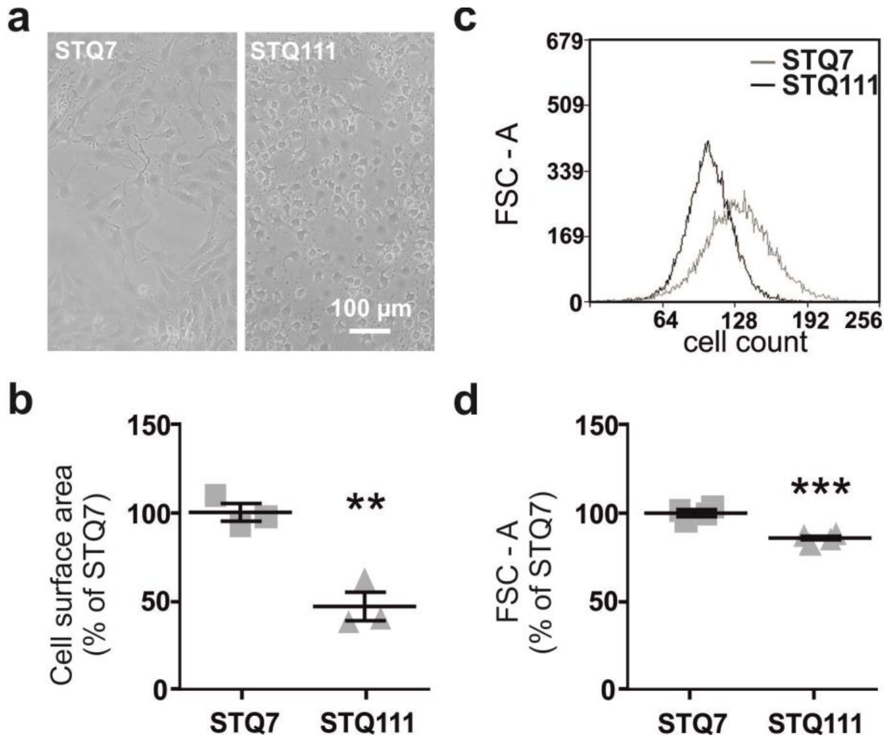

Supplementary Figure S2: Neuron- like, differentiated ST*Hdh* cells show the same cell size phenotype

(a) Representative picture of live, differentiated ST*Hdh*$^{Q7/Q7}$ (STQ7) and ST*Hdh*$^{Q111/Q111}$ (STQ111) cells and **(b)** ImageJ-based cell surface area quantification of the somata of cells, attached to the culture dish after 48 hours of differentiation; n=3 experiments, unpaired *t*-tests; ** $P < 0.01$ **(c)** Representative histograms of ST*Hdh* cells with corresponding **(d)** quantification of the cell size of live cells in suspension, based on the relative mean forward scatter area (FSC-A); n = 4 experiments, unpaired *t*-tests; *** $P < 0.001$.

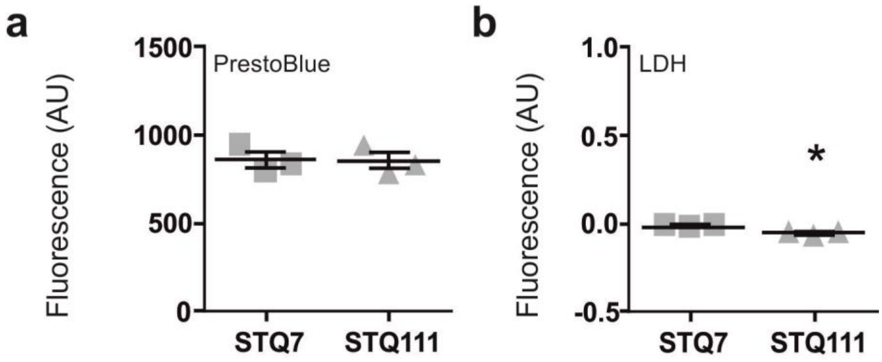

Supplementary Figure S3: Differentiation into neuron-like cells results in comparable results between ST*Hdh* and MEF*Hdh* cells

Results from the cell size- and cell number-dependent tests for 24 hours differentiated ST*Hdh* cells: **(a)** PrestoBlue®, n = 3 experiments and **(b)** LDH assay, n = 3 experiments. Unpaired *t*-tests; * $P < 0.05$.

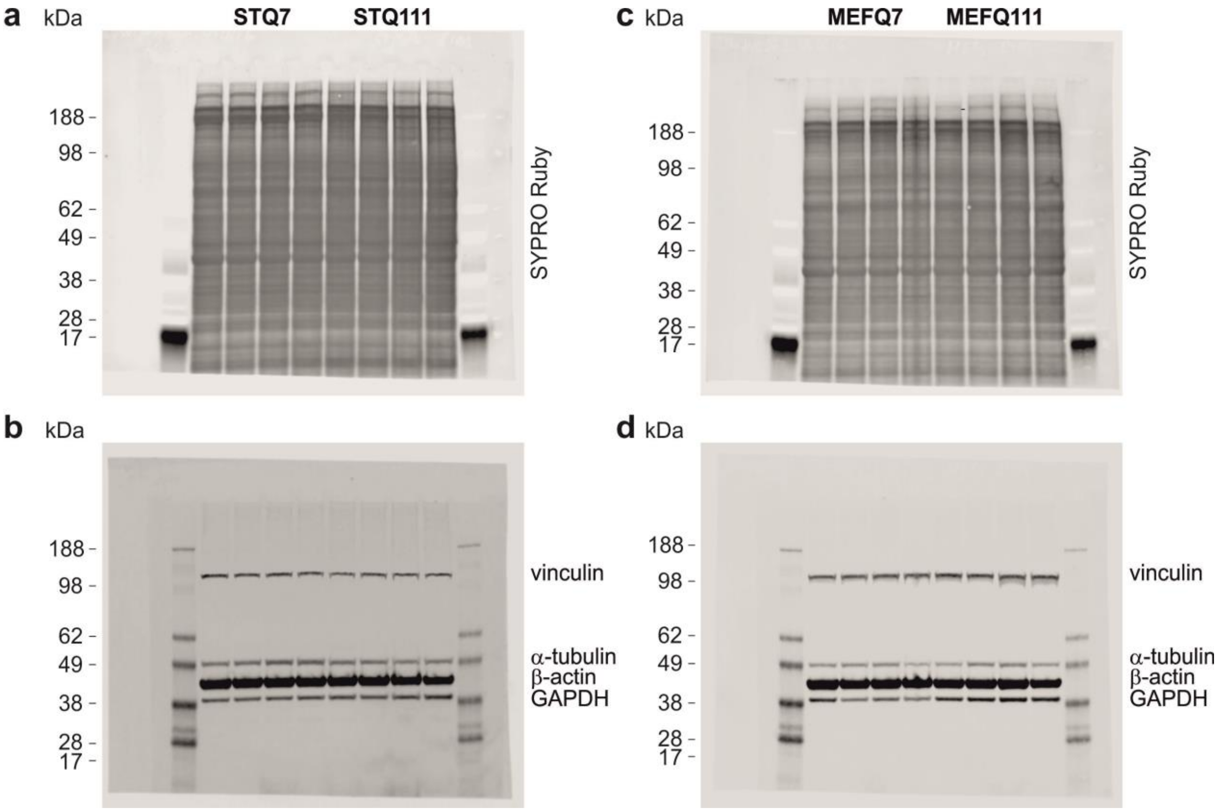

Supplementary Figure S4: Chromosomal abnormalities impede the interpretation of western blot results in ST*Hdh* cells

Full-length blots of **(a)** SYPRO Ruby staining and **(b)** analysed proteins from ST*Hdh* cell lysates **(c and d)** respective results from MEF*Hdh* cell lysates.
